# Case report: a novel approach for the emergency repair of acute aortic rupture associated with congenital aortic Coarctation

**DOI:** 10.1186/s13019-021-01552-5

**Published:** 2021-06-09

**Authors:** Xin Pu, Maozhou Wang, Xiaoyong Huang, Hongjia Zhang, Lianjun Huang

**Affiliations:** 1 Department of Intervention Diagnosis and Therapy, Beijing Anzhen Hospital, Capital Medical University, Beijing, China; 2 Department of Cardiac Surgery, Beijing Anzhen Hospital, Capital Medical University, Beijing, China

**Keywords:** Congenital aortic coarctation (CoA), Aortic rupture, Occluder, Pregnancy, Case report

## Abstract

**Background:**

Congenital aortic coarctation (CoA) associated with aortic rupture is a rare but extremely lethal condition. In pregnant patients, the condition becomes very risky.

**Case presentation:**

We presented a case of a pregnant (20 weeks gestation) patient with CoA associated with ruptured aortic pseudoaneurysm who was successfully rescued using a novel hybrid strategy.

**Conclusions:**

This hybrid approach may be a life-saving bridging intervention in patients with CoA associated with devastating complications, such as ruptured aneurysms, especially with extremely narrowed access.

## Background

Congenital aortic coarctation (CoA) is a common congenital heart disease, accounting for 5–8% of cases of congenital cardiovascular disease, and it is characterized by a narrowing of the thoracic aorta adjacent to the left subclavian artery [7, 10, 12]. Although most cases of CoA are mainly diagnosed in infancy or childhood, occasionally, the disorder may first present in adulthood, especially in hypertensive pregnant women. Here, we present a case of a 20-week pregnancy in a patient with CoA associated with ruptured aortic pseudoaneurysm who was successfully rescued using a novel hybrid strategy.

## Case presentation

### Clinical history

A 28-year-old female who was pregnant (20 weeks gestation) presented to the emergency department with complaints of the sudden onset of severe back pain and dyspnoea.

She had a history of hypertension for more than 14 years, without blood pressure control. As this was her first pregnancy, obstetrical consultations were performed regularly in a local hospital. No other abnormality was recorded, except systemic hypertension.

On emergency physical examination, her vital signs were unstable. Her heart rate was 118 beats/min. The blood pressure in her upper limbs was 80/50 mmHg, and the blood pressure in her lower limbs was too low to calculate. The breath sounds in the left lung disappeared during chest auscultation. Laboratory tests showed that her haemoglobin level had dropped to 63 g/dL.

Echocardiography revealed that the cardiac structures and function were normal, the ascending aorta was normal, and there was a massive left pleural effusion. Unfortunately, it was diagnosed as intrauterine foetal death by pelvic ultrasound.

Aortic CT angiography revealed congenital aortic coarctation associated with pseudoaneurysm formation distal to the coarcted segment with a large left-sided haemothorax. Hence, we highly suspected pseudoaneurysm rupture. Moreover, total aortic hypoplasia was observed.

### Operating procedure

Considering this patient had haemorrhagic shock, conventional open repair was deemed excessively risky. Moreover, the removal of the dead foetus as soon as possible was required to prevent secondary coagulation disorders. The patient was immediately transferred to the hybrid operating room, and general anaesthesia was performed. Right femoral artery access was established percutaneously, and a 5F sheath was inserted. A calibrated pigtail catheter (Cook Group Inc., Bloomington, Ind) was introduced through the right femoral artery and was advanced over a hydrophilic guidewire (0.035,180 cm, Radiofocus guidewire M, Terumo Europe, N.V., Leuven, Belgium) up to the ascending aorta. Left radial artery access was established to help the catheter pass through the coarcted segment. Angiography of the entire aorta was obtained, and the diagnosis was confirmed to be congenital aortic coarctation-associated aortic hypoplasia and a ruptured pseudoaneurysm distal to the coarcted segment, which caused a large left-sided haemothorax and left lung collapse.

**Fig. 1:**
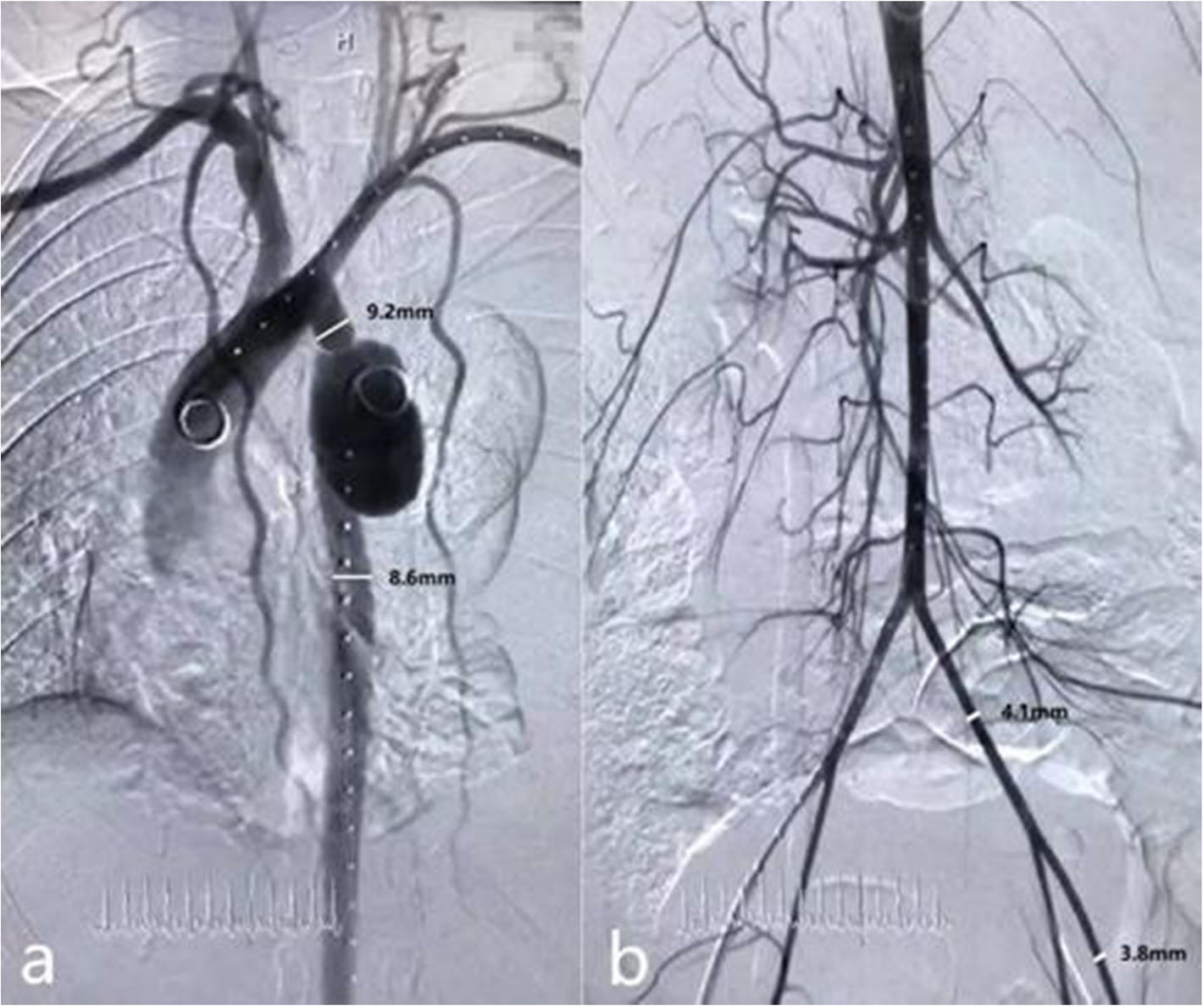
Aortic angiography. **a.** Angiogram of the thoracic aorta showed coarctation with aortic hypoplasia and a ruptured pseudoaneurysm distal to the coarcted segment. **b.** Angiogram of the abdominal aorta showed a hypoplastic aorta with extremely narrowed femoral-iliac arteries

A series of accurate measurements were performed. The diameter of the most coarcted segment was 1.8 mm, and the aortic diameters of the proximal and distal coarcted segments were only 9.2 mm and 8.6 mm, respectively. The diameters of the bilateral femoral arteries were only 3.8 mm and 3.8 mm, respectively. The diameters of the bilateral iliac arteries were only 4.1 mm, respectively (Fig. 1). The extremely narrow access to the vessel and severe aortic hypoplasia made therapeutic decisions difficult. It was impossible to perform a routine endovascular operation, such as covered-stent grafts or covered Cheatham-Platinum stent implantation (at least a 14F delivery system was required, 1F ≈ 0.33 mm).

**Fig. 2:**
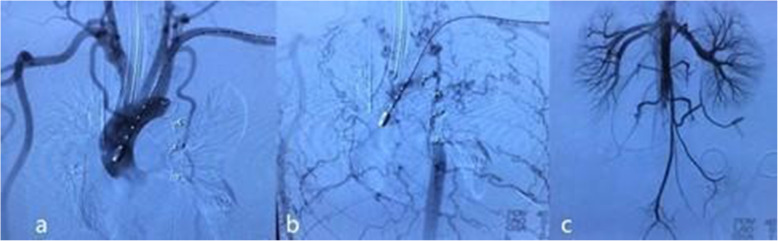
Post-operative angiography. **a.** Antegrade angiography of the ascending aorta demonstrated successful exclusion of the coarcted aortic segment and pseudoaneurysm. **b.** Antegrade angiography of the ascending aorta in the late phase revealed that the upper abdominal aorta was filled in an antegrade manner from extensive collateral circulation. **c.** Abdominal aortic angiography demonstrated that anterograde and retrograde blood flow intermixed at the distal abdominal aorta

Then, a novel approach was proposed. In the first step, a right-side axillary-femoral bypass was performed. An 8 mm * 60 mm vascular graft (GORE INTERING Vascular Graft, GORE, USA) was sutured at the right infraclavicular region, with a right femoral end-to-side anastomosis. The graft was tunneled subcutaneously along the midaxillary line to prevent graft entanglement due to torso flexion. In the second step, transcatheter aortic occlusion was performed via left femoral access. Left femoral access was obtained percutaneously, and then, a 10F long sheath (Cook, Bloomington, Indiana, USA) was advanced from the left femoral artery over a 0.035 in. superstiff guidewire (Amplatz Super Stiff, Boston Scientific, USA) through the lesion segment. Two 18/16 mm PDA occluders (Starway Medical Technology Inc., Beijing, China) were placed proximal and distal to the lesion segment to block flow. The proximal occluder was placed exactly between the origin of the left subclavian artery and the narrowest segment, and the distal occluder was placed 30 mm beyond the site of the pseudoaneurysm, where the aortic wall was morphologically normal. Postoperative angiography demonstrated patent bypass and the satisfied position of the occluders. Ruptured pseudoaneurysm was successfully excluded. No residual leakage was found. The upper abdominal aorta still filled in an anterograde manner by extensive collateral vessels. The perfusion of the abdominal visceral arteries was sufficient. The right iliac artery was filled in a retrograde manner by the bypass vessel. The anterograde and retrograde blood flows intermixed at the distal abdominal aorta (Fig. 2). After the procedure, the vital signs became stable, and the blood pressure was 125/71 mmHg in the upper limbs and 116/73 mmHg in the lower limbs. A 6F Perclose ProGlide suture closure device (Abbott Vascular, Abbott Park, Ill) was deployed at the access point. In the third step, the hysterotomy procedure was performed by an obstetrician/gynaecologist.

### Perioperative period

The patient was then transferred to the intensive care unit, and the pleural effusion was drained intermittently (300 mL every other day). She was extubated on the 3rd day after the procedure and then received low-dose antiplatelet therapy (aspirin 100 mg/daily). Her recovery was uneventful, with an upper limb blood pressure of 130/80 mmHg and a lower limb blood pressure of 120/75 mmHg. The left lung progressively re-expanded. She was discharged on the 10th day after the operation in good clinical condition.

### Follow-up

The patient was followed up for 18 months after discharge. During the follow-up, she underwent physical examination, including blood pressure (BP) measurements and CT angiography of the total aorta. Daily antiplatelet therapy was continued until now. Her blood pressure was approximately 130/70 mmHg in the upper limbs and 116/68 mmHg in the lower limbs (the pressure gradient was approximately 10–20 mmHg).

**Fig. 3:**
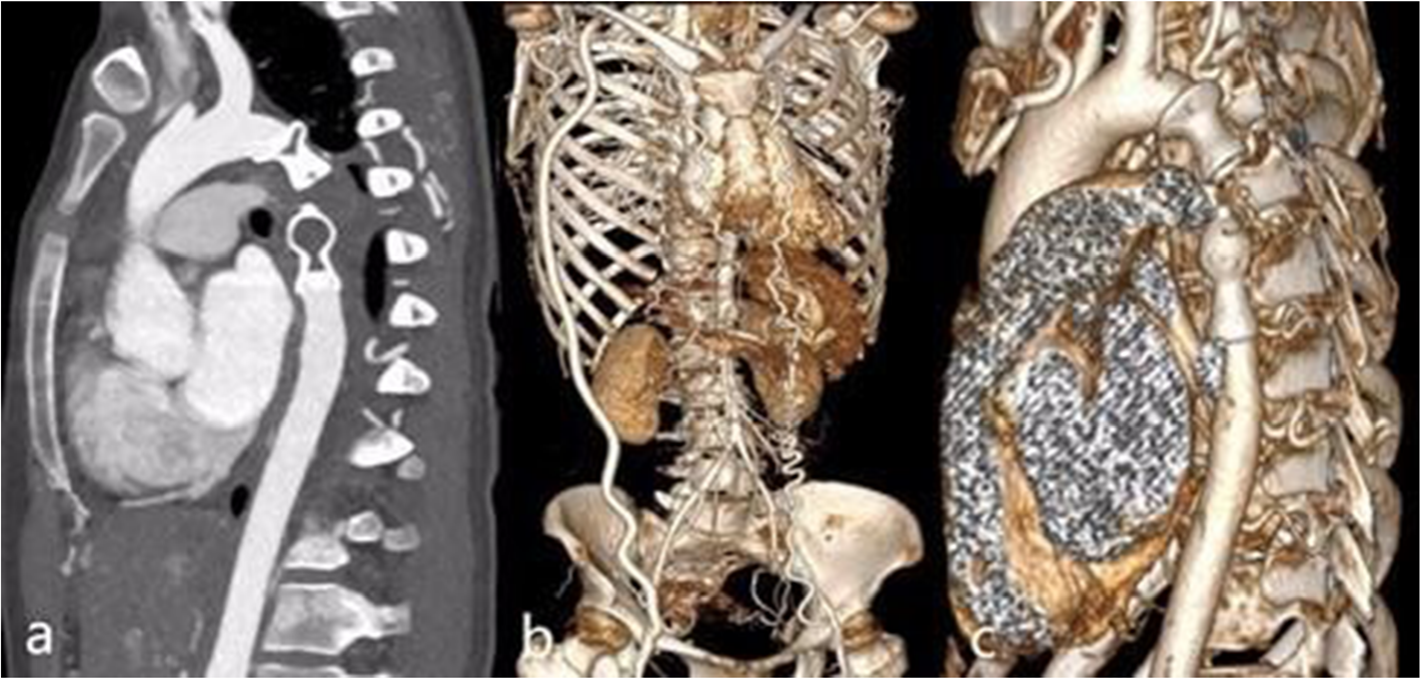
1-year follow-up CT angiography. **a**. Multiplanar reconstruction imaging (MPR) demonstrated obliteration of the coarcted segment and distal pseudoaneurysm. **b.** Three-dimensional imaging showed a patent right axillary-to-femoral bypass graft. **c**. A 3-dimensional image showed the satisfactory position and shape of the occluders

The first-year follow-up computed tomography angiography revealed that the positions of the occluders were satisfied, the coarcted segment and ruptured pseudoaneurysm were excluded completely, and the bypass vessel was patent (Fig. 3). The patient also underwent abdominal ultrasonography at the 18-month follow-up. Adequate blood flow of visceral arteries and bilateral lower limbs was revealed, and the mixed level of anterograde and retrograde blood flow was still at the distal abdominal aorta.

## Discussion and conclusion

The presentation of Congenital Coarctation of Aorta (CoA) is variable, and 15–20% of subjects remain asymptomatic until adulthood. Delayed symptoms may be due to the development of collaterals that maintain adequate flow to the lower body. Untreated CoA can result in high morbidity and mortality due to hypertension, congestive heart failure, stroke, aortic dissection, and sudden death [9, 10].

It is known that aortic medial abnormalities occur in patients with coarctation of the aorta and may develop upstream or downstream dilatation, aneurysm, or even aortic rupture. It has been reported that 32% of aneurysms are proximal to coarctation, 51% are distal, and 17% involve the LSA rather than the aorta [4].

It is known that aortic disease is more aggressive during pregnancy. The mechanism includes increased wall stress from hyperdynamic cardiovascular changes, particularly from 12 to 28 weeks gestation, and oestrogen-induced aortic media degeneration [1, 3]. Sometimes, adult CoA may be first diagnosed during pregnancy, necessitating emergency surgery [1], as in the present case.

Initially, CoA was treated with open surgery, including in situ resection and interposition graft, patch aortoplasty, subclavian flap aortoplasty, and extra-anatomical bypass [2, 8]. However, late complications are common, and the incidence of aneurysm formation following coarctation repair has been reported to be between 5 and 51% [5].

Owing to its reduced perioperative morbidity and mortality when compared with open surgery, endovascular repair has emerged as an alternative for CoA in adults, especially for CoA with aneurysm [6]. Recently, covered stents have represented an appealing option in the endovascular management of native coarctation [11, 13]. Sohrabi [10] reported that implanting covered Cheatham-Platinum stents has very high success rates with remarkable haemodynamic effects in patients with severe CoA.

However, in this case, CoA associated with a hypoplastic aorta and extremely narrowed access to vessels represented challenging conditions for treatment. Furthermore, it was an acute emergency situation. If the bleeding cannot be controlled instantly, the patient is at risk for immediate death due to circulatory collapse.

Considering the age of the patient, open aortic repair was the first-considered option. The resection and interposition graft in situ via left lateral thoracotomy was extremely risky in this condition due to the large left haemothorax. Moreover, the narrowed iliac-femoral artery could not accommodate the cardiopulmonary bypass sheath.

The endovascular management was then evaluated. However, a covered stent graft (or Cheatham-Platinum stent graft) required at least a 12F (≈4.0 mm) delivery system. Thus, the patient was not a candidate for traditional endovascular approaches due to the increased risk for injury to the access vessel and subsequent perforation.

Therefore, the novel approach of transcatheter aortic occlusion was performed. Before occlusion, an extra-anatomical axillofemoral bypass was performed. The purposes were as follows: 1, to ensure a sufficient blood supply to the abdominal organs; 2, once aortic occlusion could not be successful as expected, open repair would be the last plan that was required; and 3, this bypass vessel could be used to establish cardiopulmonary bypass. PDA occluders (2 and 18/16 mm) were implanted to occlude the ruptured aortic segment. The size of the occluder was the largest that the 10F delivery system could deliver. The procedure was successful, and postoperative aortic angiography showed an excellent occlusion effect. Because of the extensive collateral circulation, the upper abdominal aorta still filled in an anterograde manner with sufficient blood supply. The patient had uneventful follow-up.

This approach was not a one-staged radical procedure. However, due to this life-threatening condition, the first aim should be saving the patient’s life. This patient is not a simple coarctation of aorta with total aortic dysplasia. She is pregnant with a stillbirth. She was in poor condition because of massive blood loss before operation. We performed aortic occlusion as soon as possible to save lives. But then we need to clear the stillbirth, otherwise disseminated intravascular coagulation and other serious complications may occur. We consider that the risk of radical surgery immediately at that time is too high, so we plan to perform radical surgery in the future. In addition, according to our postoperative angiography, all abdominal organs have sufficient blood supply by means of collateral circulation, Anterograde blood flow was presented in the abdominal aorta. Considering the patient’s overall fitness and the pressure gradient between the upper and lower limbs being less than 20 mmHg during the follow-up, no further surgery was planned in the short term. At present, the patients were followed up for 2 years, and the general condition was good. However, a strict follow-up plan was needed. Once the pressure gradient between the upper and lower limbs being more than 20 mmHg or the appearance of abdominal organ ischemia during the follow-up, the radical surgery will be performed as soon as possible.

Patients feltel that they have had a good quality of life after the operation. The patient did not feel any discomfort related to operation during the 2 year follow up.

To the best of our knowledge, this is the first case of a successful rescue in a patient with CoA associated with a ruptured pseudoaneurysm reported worldwide, not to mention in a pregnant patient. This hybrid approach may be a life-saving bridging intervention in patients with CoA associated with devastating complications, such as a ruptured aneurysm, especially with extremely narrowed access.

## Data Availability

*The data underlying this article cannot be shared publicly due to* the privacy of the individuals who participated in the study. *The data will be shared on reasonable request to the corresponding author.*

## Abbreviations

CoA: Congenital aortic coarctation
BP: Blood pressure

## References

[1] Anass A, Pascal S, Alain F (2013). Transcatheter therapy for aortic coarctation with severe systemic hypertension during pregnancy. Catheter Cardiovasc Interv.

[2] Charlton-Ouw KM, Codreanu ME, Leake SS, Sandhu HK, Calderon D, Azizzadeh A, Estrera AL, Safi HJ (2015). Open repair of adult aortic coarctation mostly by a resection and graft replacement technique. J Vasc Surg.

[3] Crawford JD, Hsieh CM, Schenning RC, Slater MS, Landry GJ, Moneta GL (2016). Genetics, pregnancy, and aortic degeneration. Ann Vasc Surg.

[4] Di Tommaso L, Mannacio VA, Di Tommaso E, Pinna GB, Fontana I, Iannelli G (2017). Endovascular treatment of distal aortic arch aneurysm associated with Coarctation of aorta in a Jehovah's witness. Tex Heart I J.

[5] Erik B, Jassar Arminder S (2018). Coarctation repair-redo challenges in the adults: what to do?. J Vis Surg.

[6] Galiñanes EL, Krajcer Z (2018). Endovascular treatment of coarctation and related aneurysms. J Cardiovasc Surg.

[7] Hoffman JIE, Kaplan S (2002). The incidence of congenital heart disease. J Am Coll Cardiol.

[8] Kouchoukos NT, Scharff JR, Castner CF (2015). Repair of primary or complicated aortic coarctation in the adult with cardiopulmonary bypass and hypothermic circulatory arrest. J Thorac Cardiovasc Surg.

[9] Lala S, Scali ST, Feezor RJ, Chandrekashar S, Giles KA, Fatima J, Berceli SA, Back MR, Huber TS, Beaver TM, Beck AW (2018). Outcomes of thoracic endovascular aortic repair in adult coarctation patients. J Vasc Surg.

[10] Sohrabi B, Jamshidi P, Yaghoubi A, Habibzadeh A, Hashemi-aghdam Y, Moin A (2014). Comparison between covered and bare Cheatham-platinum stents for endovascular treatment of patients with native post-ductal aortic Coarctation: immediate and intermediate-term results. J Am Coll Cardiol Intv.

[11] Suárez De Lezo J, Romero M, Pan M, Suárez De Lezo J, Segura J, Ojeda S (2015). Stent Repair for Complex Coarctation of Aorta. J Am Coll Cardiol Intv.

[12] Taggart NW, Minahan M, Cabalka AK, Cetta F, Usmani K, Ringel RE (2016). Immediate outcomes of covered stent placement for treatment or prevention of Aortic Wall injury associated with Coarctation of the aorta (COAST II). J Am Coll Cardiol Intv.

[13] Tanous D, Collins N, Dehghani P, Benson LN, Horlick EM (2010). Covered stents in the management of coarctation of the aorta in the adult: initial results and 1-year angiographic and hemodynamic follow-up. Int J Cardiol.

